# Supplementary material for: Inferring collective dynamical states from widely unobserved systems
Source: Nat Commun. 2018 Jun 13;9:2325. doi: 10.1038/s41467-018-04725-4 (PMC5998151; doi:10.1038/s41467-018-04725-4)
Supplement: Supplementary file 1 — Supplementary Information [file 41467_2018_4725_MOESM1_ESM.pdf]

Supplementary Information for

Inferring collective dynamical states from widely unobserved systems

J. Wilting[1] & V. Priesemann[1,2,*]

[1]Max-Planck-Institute for Dynamics and Self-Organization, Göttingen, Germany; [2]Bernstein-Center for Computational Neuroscience, Göttingen, Germany
[*] viola@nld.ds.mpg.de

19 pages:

- Supplementary Figures 1 – 7 (7 pages)
- Supplementary Table 1 (1 page)
- Supplementary Notes 1 – 10 (8 pages)
- Supplementary References

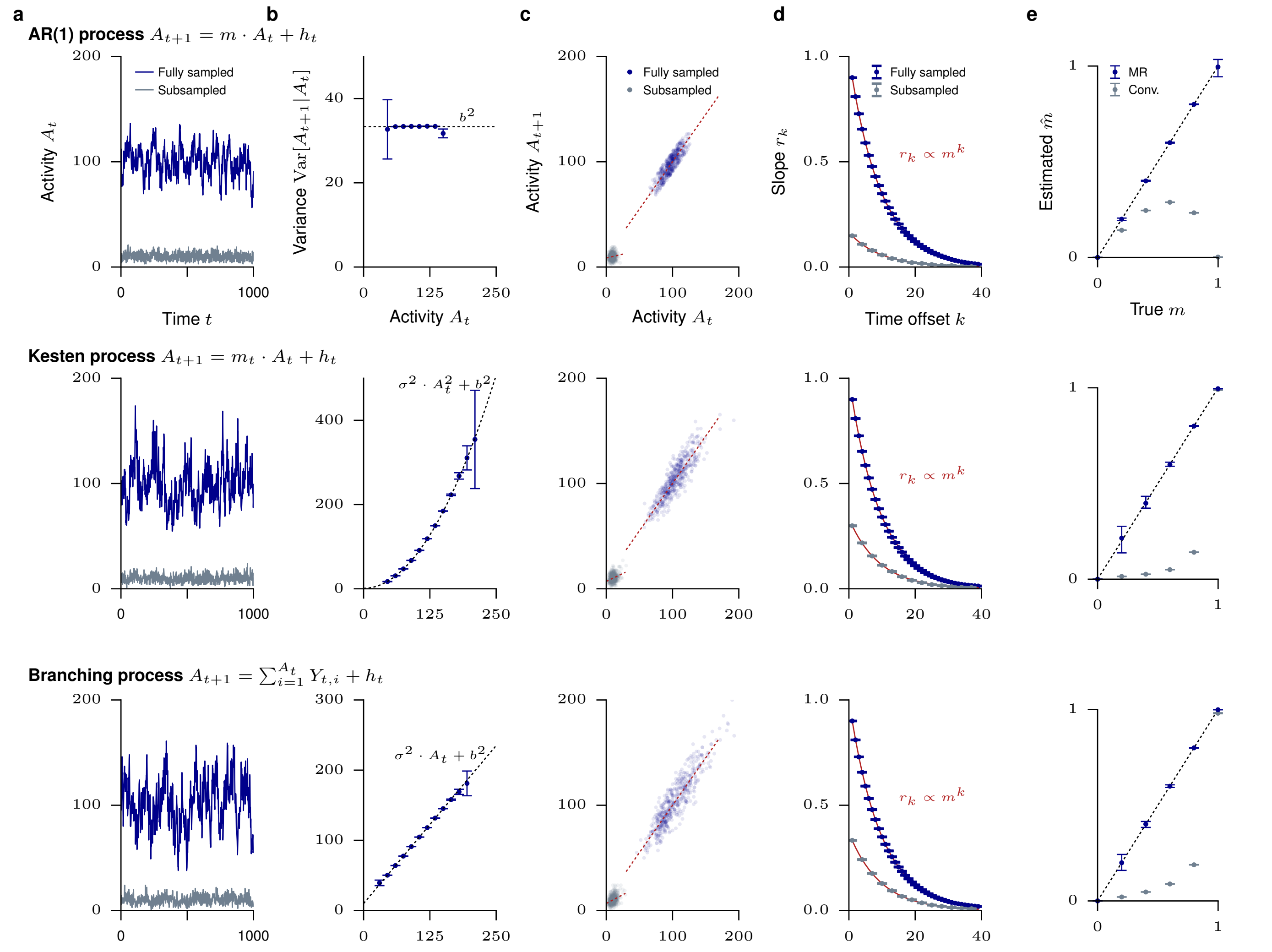

Supplementary Figure 1: **MR estimation for PARs.** Although derived for branching processes (BPs), we conjectured that MR estimation is applicable to any process with a first order autoregressive representation (PAR). We here show exemplary results for three different classes of PARs: In AR(1) processes, additive noise h_t is drawn independently at each time step. Here, we considered a uniform distribution $h_t \sim \mathscr{U}(0, 2h)$. In a Kesten process, additive and multiplicative noise is drawn at each time step, both m_t and h_t being i.i.d. for all t. Here, $m_t \sim \mathscr{N}(m, \sigma^2)$ with $\sigma = m/10$ and $h_t \sim \mathscr{N}(h, b^2)$ with $b = h/10$ are normally distributed. In a BP, each unit i at time t generates $Y_{t,i}$ offspring, which are i.i.d. for all t and i. In addition, a random number h_t of units are introduced at each time step. Here, $Y_{t,i} \sim \mathrm{Poi}(m)$ and $h_t \sim \mathrm{Poi}(h)$ are Poisson distributed, σ^2 and b^2 denote the variances of $Y_{t,i}$ and h_t respectively. All three processes satisfy the first-order statistical recursion relation $\langle A_{t+1} | A_t \rangle = mA(t) + h$ (Eq. (5)). Parameters are chosen such that for all simulations the average activity is identical, $\langle A_t \rangle = 100$. **a**. Fully sampled and subsampled (binomial subsampling $a_t \sim \mathrm{Bin}(A_t, \alpha)$ with $\alpha = 1/10$) time series are shown for $m = 0.9$ and $h = 10$. **b**. The three classes show the same first-order statistics according to Eq. (5). However, their second order statistics $\mathrm{Var}[A_{t+1} | A_t]$ differ as indicated. **c**. Conventional linear regression underestimates $\hat{m}$ for all three processes under subsampling. **d**. MR estimation is applicable to all three processes under full sampling and subsampling, i.e. $r_k \propto m^k$ holds. **e**. While MR estimation returns consistent estimates of m even under subsampling, the conventional estimator underestimates $\hat{m}$ for all three processes.

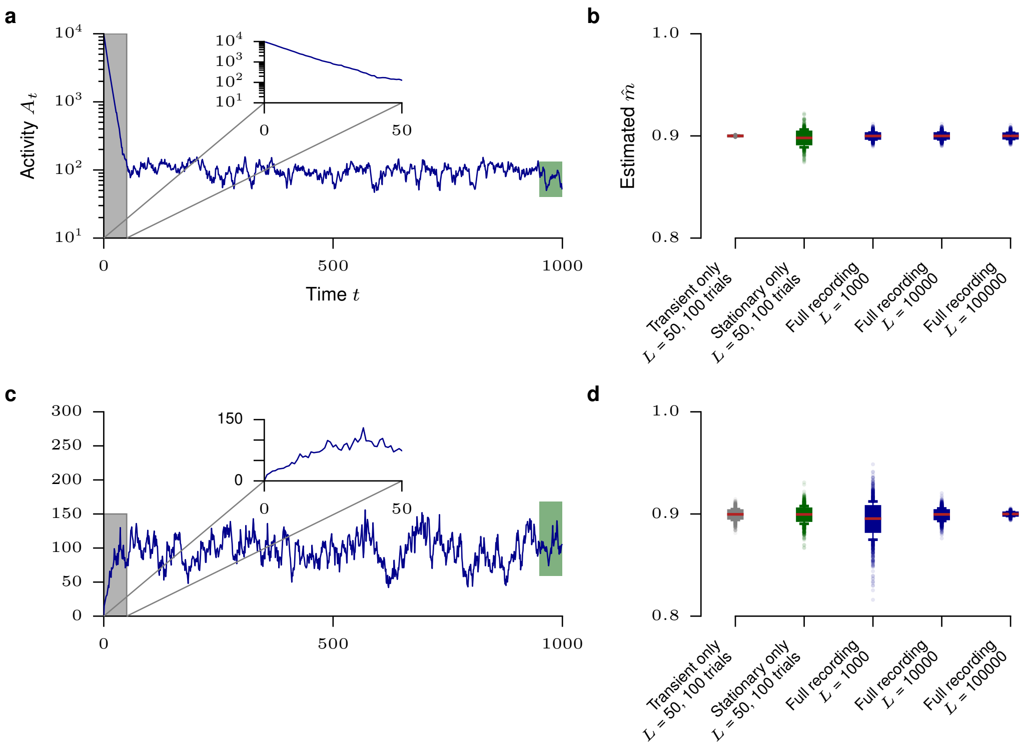

Supplementary Figure 2: MR estimation with transients. A branching process (BP) with $m = 0.9$ and expected activity $\langle A_t \rangle = 100$ is started far from the stationary distribution, namely with $A_0 = 10,000$ (top) or $A_0 = 0$ (bottom). Using MR estimation, $\hat{m}$ is inferred from: (i) only the first 50 data points of 100 independent trials, i.e. only transient parts of the activity in each trial (gray); (ii) 50 data points of 100 independent trials after the activity was allowed to relaxate to the stationary distribution in each trial (green); (iii) from one single trial comprising both transient and stationary parts, using 10^3, 10^4, or 10^5 time steps (blue). **a**, **c**. Activity A_t of one single trial of 10^3 time steps as a function of time t. Insets show magnified transient period where A_t converges to the stationary distribution. Shaded areas indicate transient (gray) and stationary (green) parts taken into account for estimates (i) and (ii) respectively. **b**, **d**. Boxplots (derived from 1000 independent realizations) for the result $\hat{m}$ of MR estimation, based on the data specified above.

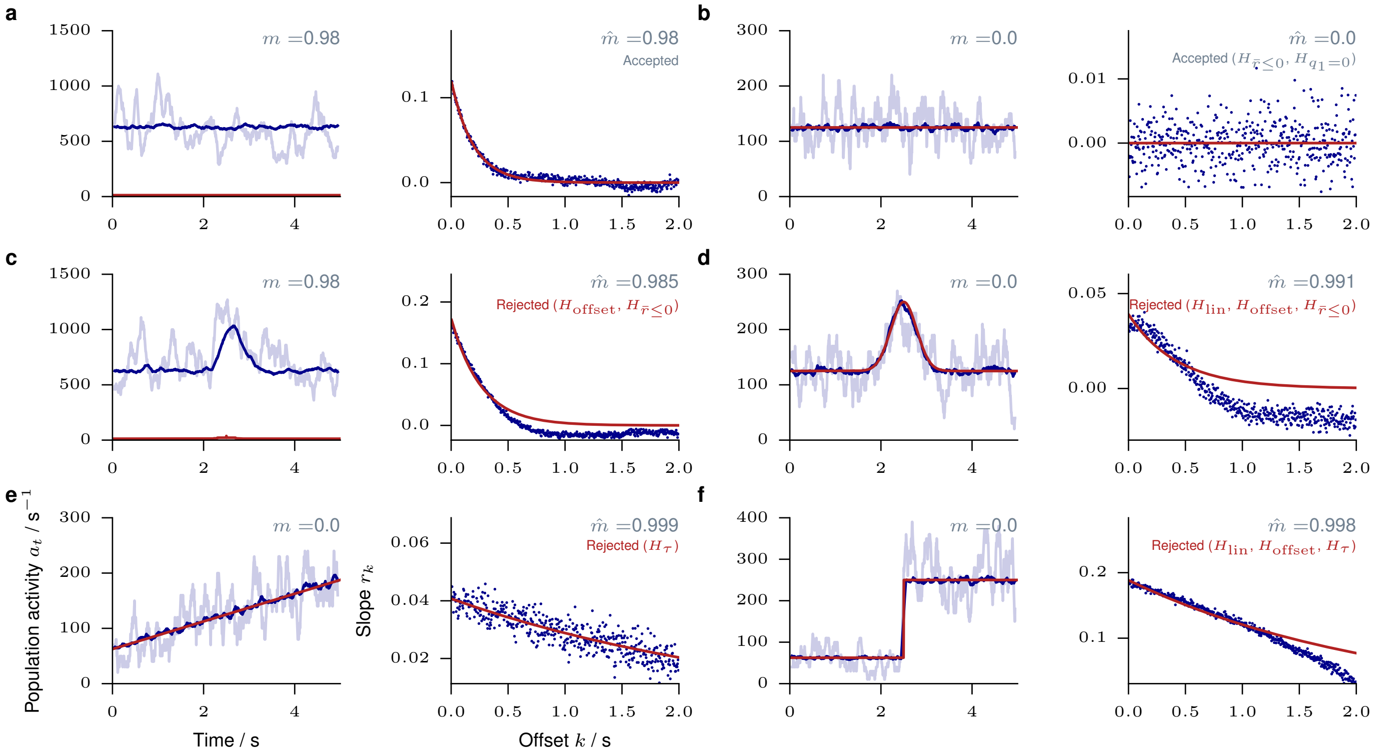

Supplementary Figure 3: Excluding nonstationary data. Each left panels shows the time series a_t of the activity from one single trial (light blue) and averaged activity from 100 trials (dark blue), recorded from $n = 50$ out of $N = 10^4$ neurons. Each right panels shows the corresponding MR estimation from one single trial. We investigated the following, generic cases for the temporal evolution of the drive rate $\langle h_t \rangle$: **a**, **b**. The drive is stationary ($\langle h_t \rangle$ identical for all t, red), so are the mean rates $\langle a_t \rangle$. **c**, **d**. The drive exhibits a transient increase centered around half of the simulation time. The mean rate $\langle a_t \rangle$ is therefore also time-dependent and follows the temporal evolution of $\langle h_t \rangle$. **e**. The drive shows a linear increase over the simulation. **f**. The drive exhibits a step function after half the simulation. Nonstationarities (**c** – **f**) typically lead to an overestimation of $\hat{m}$, which is particularly severe if the underlying dynamics is Poissonian ($m = 0$). The tests defined in Supplementary Note 5 (see Supplementary Table 1) were able to exclude time series where the investigated nonstationarities were present, while accepting the stationary cases **a**, **b**.

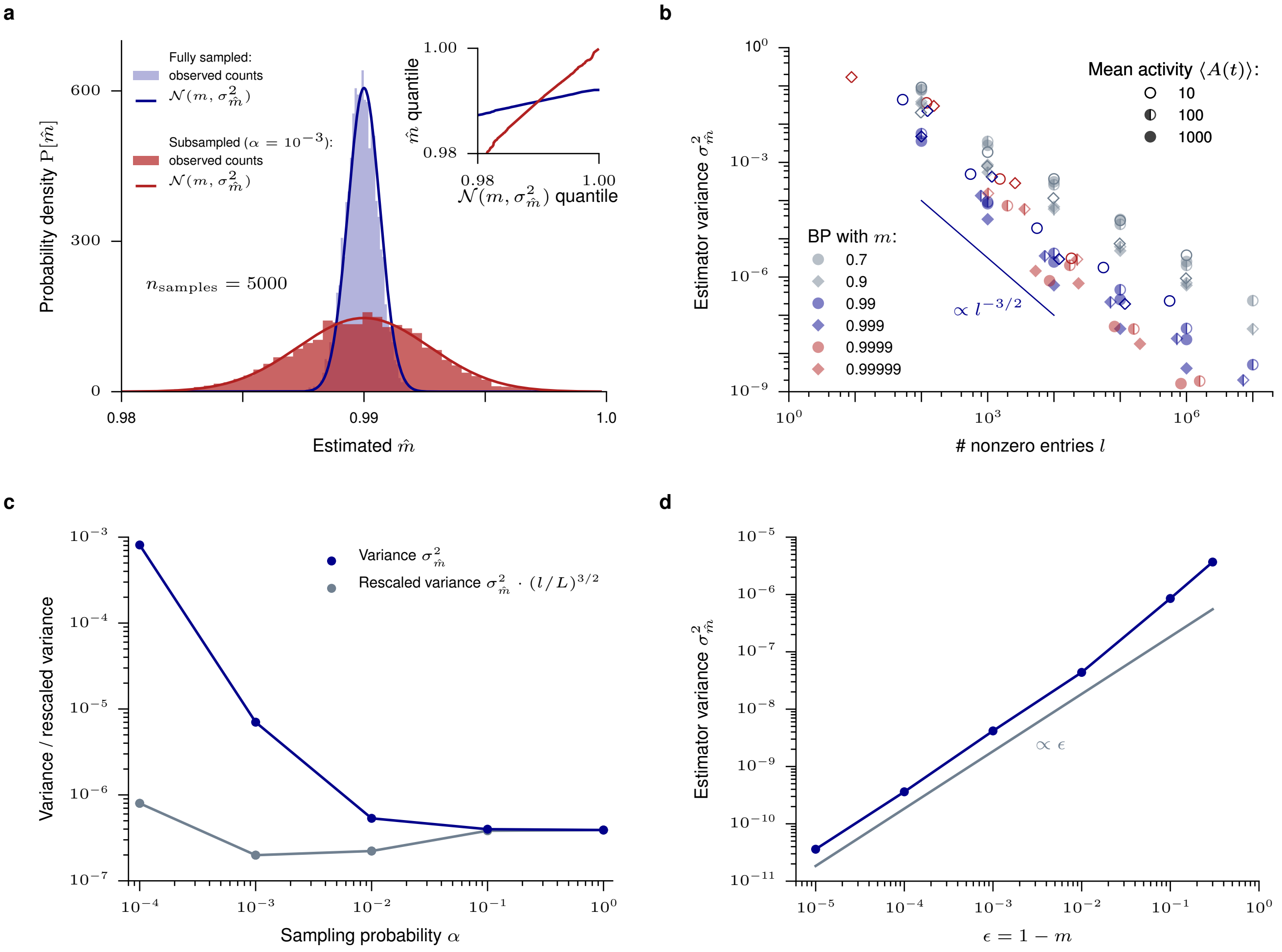

Supplementary Figure 4: Variance of the MR estimates. This figure shows numerical result for the distribution and variability of the estimate $\hat{m}$ as a function of multiple parameters. **a.** Distribution of the estimate $\hat{m}$, estimated from 5000 independent copies of a branching process (BP) with $m = 0.99$, $\langle A_t \rangle = 100$ and length $L = 10^5$: normalized histograms of the probability of estimating $\hat{m}$ for full sampling (blue) and binomial subsampling with $\alpha = 0.001$ (red), together with normal distributions $\mathcal{N}(m, \hat{\sigma}^2_{\hat{m}})$. Inset: Q-Q-plot for the quantiles of $\mathcal{N}(m, \hat{\sigma}^2_{\hat{m}})$ and the quantiles of the estimated $\hat{m}$ under both samplings. The estimated $\hat{m}$ are found to be distributed normally in both cases (fully sampled: $r^2 = 0.9995$, subsampled: $r^2 = 0.998$). **b.** The variance $\sigma^2_{\hat{m}}$ of the estimate $\hat{m}$ is estimated from 100 independent copies of a BP. Results for different m, mean activities $\langle A_t \rangle$ and time series lengths L are plotted as a function of the effective time series length $l = |\{A_t | A_t > 0\}|$, the number of nonzero entries. For any given m, the variance of $\hat{m}$ shows algebraic scaling $\sigma^2_{\hat{\epsilon}} \propto l^{\gamma}$. The exponent of this scaling depends on m, with higher γ the closer m is to unity. Hence, the benefit from longer time series is larger the closer a system is to criticality. Importantly, the variance does not directly depend on the mean activity $\langle A_t \rangle$, this number only influences the accuracy of MR estimation via the potential change in l. **c.** The variance of the estimate $\hat{m}$ is estimated from 100 independent copies of a BP with $m = 0.99$, $\langle A_t \rangle = 100$, and $L = 10^5$ and plotted as a function of the sampling probability α under binomial subsampling. While the variance appears to increase dramatically under stronger subsampling, this increase can be attributed to the according decrease of the effective time series length l. After rescaling by $(l/L)^{3/2}$ (cf. panel **b**), the rescaled variance remains within one order of magnitude over four orders of magnitude in α. Hence, the accuracy of the estimator is not directly influenced by the degree of subsampling. **d.** The variance $\sigma^2_{\hat{m}}$ is estimated from 100 independent copies of a BP with $m = 0.99$, $h = 1$, and $L = 10^5$ and plotted as function of the distance to criticality $\epsilon = 1 - m$. The variance is found numerically to scale as $\sigma^2_{\hat{m}} \propto \epsilon$, hence the standard deviation scales as $\sigma_{\hat{m}} \propto \sqrt{\epsilon}$. Similar scaling results were found for other linear (like the interquartile range) and quadratic (like the mean squared error) measures of variation.

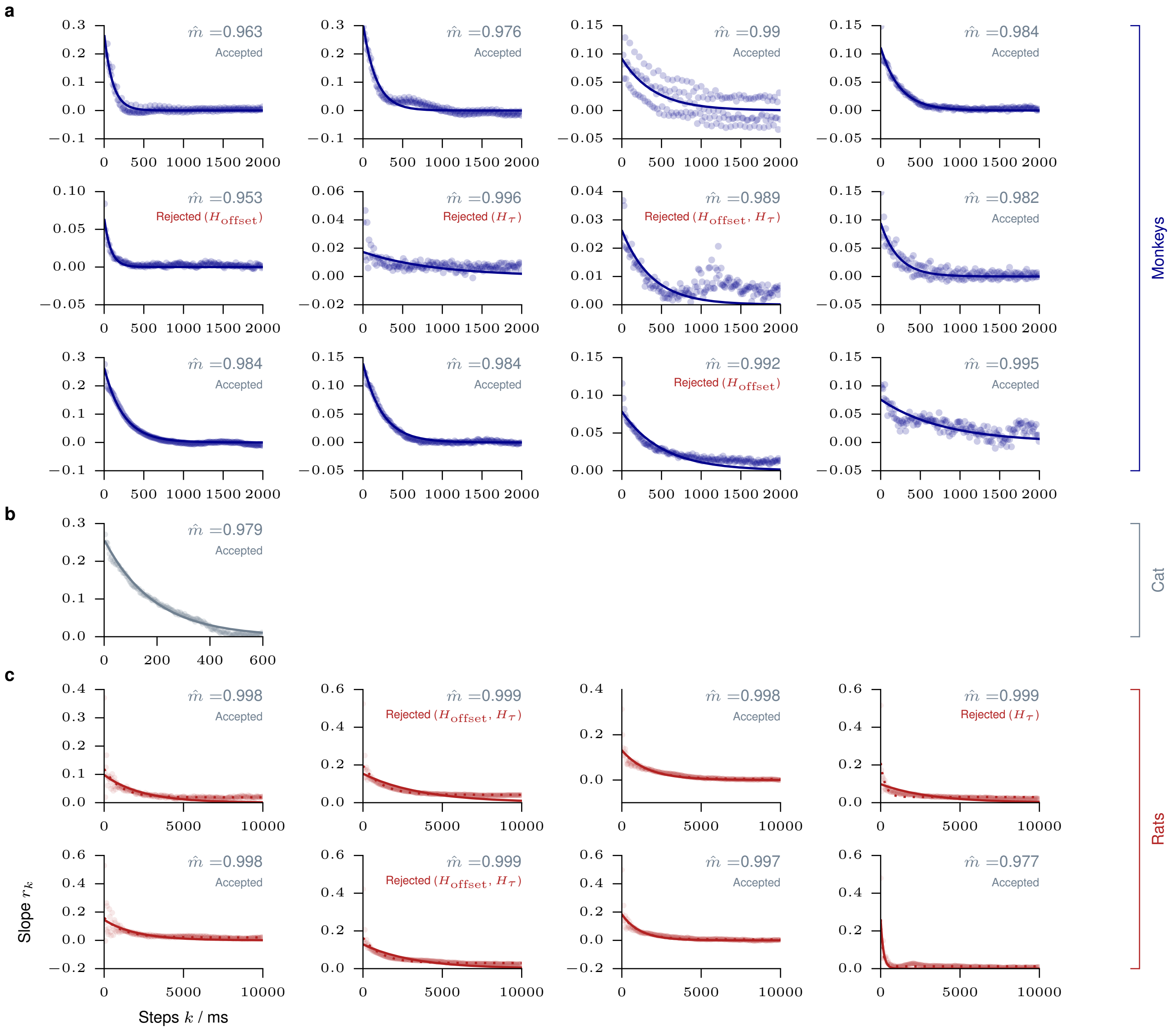

Supplementary Figure 5: **MR estimation for individual animals**. MR estimation is shown for every individual animal (see Supplementary Note 10). The consistency checks are detailed in the Supplementary Note 5 (see Supplementary Table 1). **a**. Data from monkey prefrontal cortex during an working memory task. The third panel shows a oscillation of r_k with a frequency of 50 Hz, corresponding to measurement corruption due to power supply frequency. **b**. Data from anesthetized cat primary visual cortex. **c**. Data from rat hippocampus during a foreaging task. In addition to a slow exponential decay, the slopes r_k show the ϑ-oscillations of 6 – 10 Hz present in hippocampus. Dashed lines indicate results for an exponential model with offset, solid lines results for the model without offset (compare Supplementary Note 5).

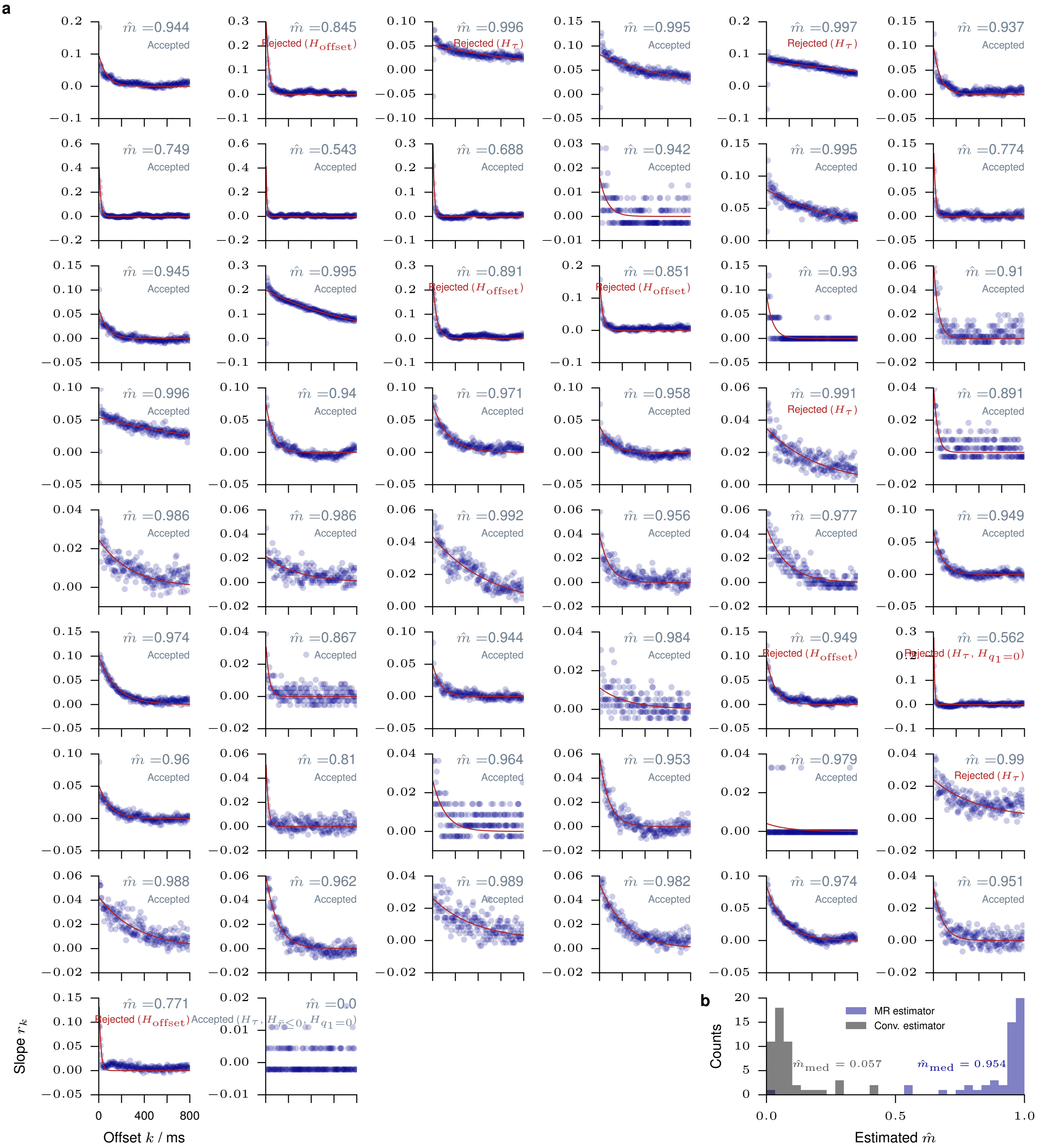

Supplementary Figure 6: MR estimation from single neuron activity (cat). MR estimation is used to estimate $\hat{m}$ from the activity a_t of a single neurons in cat visual cortex. **a**. Each panel shows MR estimation for one of the 50 recorded neurons. Autocorrelations decay rapidly in some neurons, but long-term correlations are present in the activity of most neurons. The consistency checks are detailed in Supplementary Note 5 (see Supplementary Table 1). **b**. Histogram of the single neuron branching ratios $\hat{m}$, inferred with the conventional estimator and using MR estimation. The difference between these estimates demonstrates the subsampling bias of the conventional estimator, and how it is overcome by MR estimation.

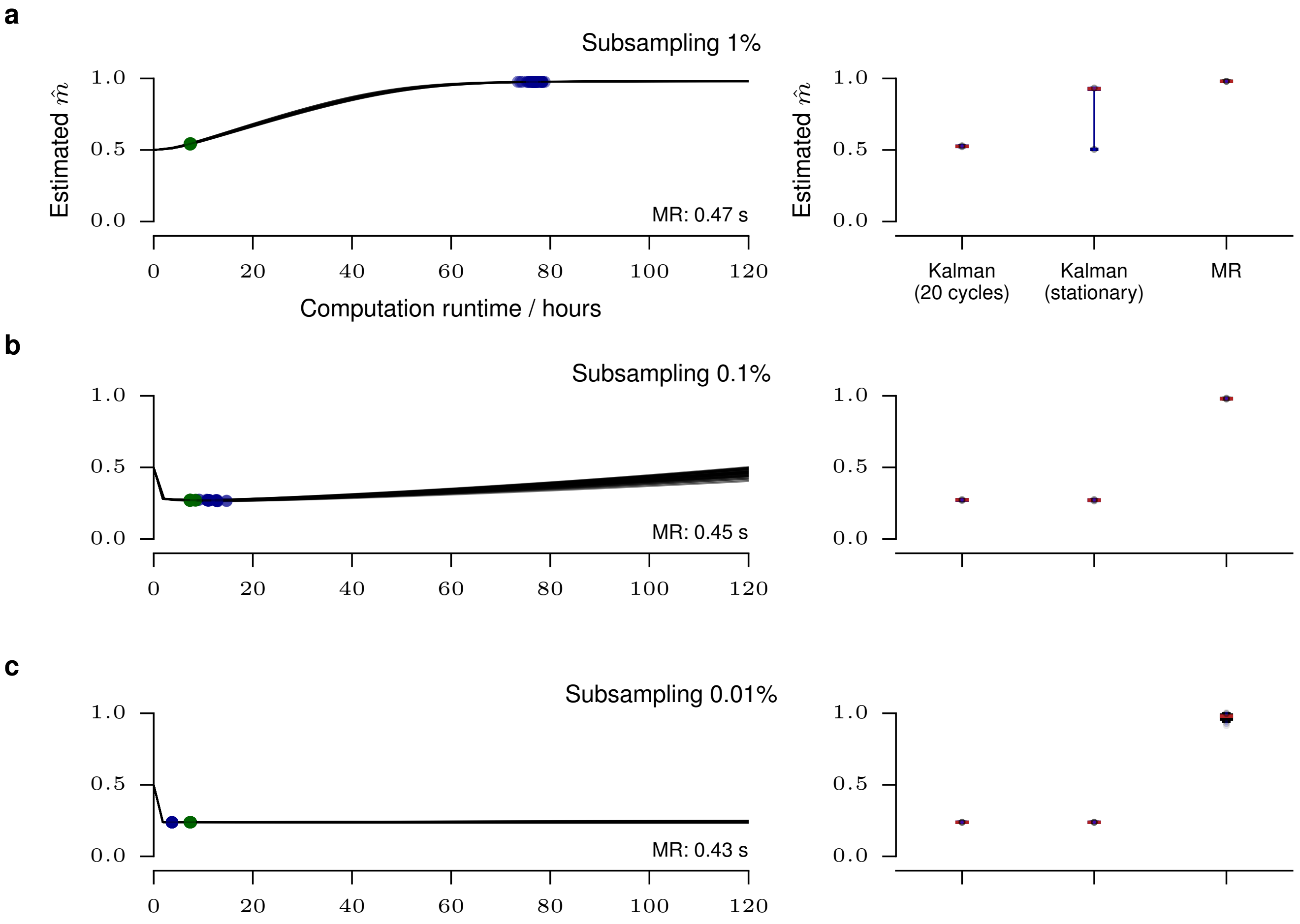

Supplementary Figure 7: Kalman EM estimation. Expectation maximization (EM) based on Kalman filtering and MR estimation are used to infer $\hat{m}$ from BPs with $m = 0.99$ and different degrees of subsampling. Left column: inferred $\hat{m}$ as a function of the EM runtime for 100 independent copies of the BP. The EM algorithm is terminated after 20 cycles (green dots) or after the inferred $\hat{m}$ changed only marginally (blue dots, see Supplementary Note 7). The median runtime of MR estimation for the same BPs is also indicated. Right column: estimated $\hat{m}$ for all three methods. **a**. Under 1% subsampling, the EM algorithm converged after runtimes of about 80 h, compared to 0.43 s for MR estimation. **b**. Under 0.1% subsampling, $\hat{m}$ inferred by the EM algorithm reaches a steady state after 10 h, but is severely biased. The slow rise of $\hat{m}$ might lead to a convergance to the proper m after several weeks of projected runtime (ignoring common termination criteria). **c**. Under 0.01% subsampling, $\hat{m}$ inferred by the EM algorithm converge to a biased value. In contrast, MR estimation returns a correct $\hat{m}$ in all three cases, and outperforms the EM algorithm by a factor of 10^5 to 10^6 in terms of the runtime.

H_{offset}	H_{τ}	H_{lin}	$H_{\tilde{r}\leqslant 0}$	$(H_{q_1=0})$	interpretation	
×	×	×	×	–	BP with $m = \hat{m}$ explains data	MR estimation valid
✓	–	–	–	–	data not explained by BP	MR estimation invalid
–	✓	–	–	–		
–	–	✓	–	–		
–	–	–	✓	×		
–	–	–	✓	✓	Poisson activity ($m = 0$) explains data	MR estimation valid

Supplementary Table 1: **Consistency checks for MR estimation.** In order to assess if the results obtained from MR estimation are consistent with a BP with stationary parameters, we perform five tests (Supplementary Note 5). We discriminate the following cases in this order: A BP with $m = \hat{m}$ is only considered to explain the data, if the four tests H_{offset}, H_{τ}, H_{lin}, and $H_{\tilde{r}\leqslant 0}$ are negative (×). If any of H_{offset}, H_{τ}, or H_{lin} is positive (✓), the data cannot be explained by a BP with any m, regardless of the other tests (–), and MR estimation is invalid. If $H_{\tilde{r}\leqslant 0}$ is positive, the additional test $H_{q_1=0}$ becomes relevant: if it is negative, the data cannot be explained by a BP with any m. If it is also positive, the data are consistent with Poisson activity (BP with $m = 0$).

Supplementary Note 1 Applicability of MR estimation

We here analytically derive the novel MR estimator for branching processes (BP)[1–3]. We expect that analogous derivations apply to any process with a first order autoregressive representation (PAR)[4], because these processes fulfill Eq. (5). Beside BPs, PARs include autoregressive AR(1) processes, integer-valued autoregressive INAR(1) processes[5] rounded integer-valued autoregressive RINAR(1) processes[6], and Kesten processes[7].

We emphasize that the MR estimator only requires the subsampled recording a_t of a system with full activity A_t conforming with the definition below. It is not necessary to know either the full system size, the number of subsampled units, nor any of the moments of the full process A_t.

Supplementary Note 2 Branching processes

In a branching process (BP) with immigration[1–3] each unit i produces a random number $y_{t,i}$ of units in the subsequent time step. Additionally, in each time step a random number h_t of units immigrates into the system (drive). Mathematically, BPs are defined as follows[1,2]: Let $y_{t,i}$ be independently and identically distributed non-negative integer-valued random variables following a law $\mathscr{Y}$ with mean $m = \langle \mathscr{Y} \rangle$ and variance $\sigma^2 = \mathrm{Var}[\mathscr{Y}]$. Further, $\mathscr{Y}$ shall be non-trivial, meaning it satisfies $\mathrm{P}[\mathscr{Y} = 0] > 0$ and $\mathrm{P}[\mathscr{Y} = 0] + \mathrm{P}[\mathscr{Y} = 1] < 1$. Likewise, let h_t be independently and identically distributed non-negative integer-valued random variables following a law $\mathscr{H}$ with mean rate $h = \langle \mathscr{H} \rangle$ and variance $\xi^2 = \mathrm{Var}[\mathscr{H}]$. Then the evolution of the BP A_t is given recursively by

$$A_{t+1} = \sum_{i=1}^{A_t} y_{t,i} + h_t, \tag{1}$$

i.e. the number of units in the next generation is given by the offspring of all present units and those that were introduced to the system from outside.

The stability of BPs is solely governed by the mean offspring m. In the subcritical state, $m < 1$, the population converges to a stationary distribution A_∞ with mean $\langle A_\infty \rangle = h/(1-m)$. At criticality ($m = 1$), A_t asymptotically exhibits linear growth, while in the supercritical state ($m > 1$) it grows exponentially. We will first show results that further specify the mean and variance of subcritical branching processes.

Theorem 1. *The stationary distribution of a subcritical BP satisfies*

$$\langle A_\infty \rangle = \frac{h}{1-m}, \qquad \mathrm{Var}[A_\infty] = \frac{1}{1-m^2}\left(\xi^2 + \sigma^2 \frac{h}{1-m}\right),$$

where m, σ^2, h, and ξ^2 are defined as above.

Proof. The first result was stated before[2,8] and follows from taking expectation values of both sides of Eq. (1): $\langle A_{t+1} \rangle = m\langle A_t \rangle + h$. Because of stationarity $\langle A_{t+1} \rangle = \langle A_t \rangle = \langle A_\infty \rangle$ and the result follows easily. For the second result, observe that by the theorem of total variance, $\mathrm{Var}[A_{t+1}] = \langle \mathrm{Var}[A_{t+1} \,|\, A_t] \rangle + \mathrm{Var}[\langle A_{t+1} \,|\, A_t \rangle]$, where $\langle \cdot \rangle$ denotes the expected value, and $A_{t+1} \,|\, A_t$ conditioning the random variable A_{t+1} on A_t. Because A_{t+1} is the sum of independent random variables, the variances also sum: $\mathrm{Var}[A_{t+1} \,|\, A_t] = \sigma^2 A_t + \xi^2$. Using the result for $\langle A_\infty \rangle$ one then obtains

$$\mathrm{Var}[A_{t+1}] = \xi^2 + \sigma^2 \frac{h}{1-m} + \mathrm{Var}[mA_t + h] = \xi^2 + \sigma^2 \frac{h}{1-m} + m^2 \mathrm{Var}[A_t]. \tag{2}$$

Again, in the stationary distribution $\mathrm{Var}[A_{t+1}] = \mathrm{Var}[A_t] = \mathrm{Var}[A_\infty]$ and hence the stated result follows. □

Supplementary Note 3 Subsampling

To derive the MR estimator for subsampled data, subsampling is implemented in a parsimonious way, according to the following definition:

Definition 1 (Subsampling). Let $\{A_t\}_{t \in \mathbb{N}}$ be a BP and $\{a_t\}_{t \in \mathbb{N}}$ a sequence of random variables. Then $\{a_t\}_{t \in \mathbb{N}}$ is called a subsampling of $\{A_t\}_{t \in \mathbb{N}}$ if it fulfills the following three conditions:

(i) Let $t', t \in \mathbb{N}$, $t' \neq t$. Then the conditional random variables[†] $(a_t | A_t = j)$ and $(a_{t'} | A_{t'} = l)$ are independent for any outcome $j, l \in \mathbb{N}$ of $A_t, A_{t'}$. If $A_t = A_{t'}$ then $(a_t | A_t = j)$ and $(a_{t'} | A_{t'} = j)$ are identically distributed.

(ii) Let $t \in \mathbb{N}$. Conditioning on a_t does not add further information to the process: The two random variables $(A_{t+1} \,|\, A_t = j, a_t = l)$ and $(A_{t+1} \,|\, A_t = j)$ are identically distributed for any $j, l \in \mathbb{N}$.

(iii) There are constants $\alpha, \beta \in \mathbb{R}$, $\alpha \neq 0$, such that $\langle a_t \,|\, A_t = j \rangle = \alpha j + \beta$ for all $t, j \in \mathbb{N}$.

Thus the subsample a_t is constructed from the full process A_t based on the three assumptions: (i) The sampling process does not interfere with itself, and does not change over time. Hence the realization of a subsample at one time does not influence the realization of a subsample at another time, and the conditional *distribution* of $(a_t|A_t)$ is the same as $(a_{t'}|A_{t'})$ if $A_t = A_{t'}$. However, even if $A_t = A_{t'}$, the subsampled a_t and $a_{t'}$ do not necessarily take the same value. (ii) The subsampling does not interfere with the evolution of A_t, i.e. the process evolves independent of the sampling. (iii) *On average* a_t is proportional to A_t up to a constant term.

It will be shown later, that the novel estimator is applicable to any time series a_t that was acquired from a BP conforming with this definition of subsampling. We will demonstrate possible applications at the hand of two examples:

1. Diagnosing infections with probability α. For example, when a BP A_t represents the spread of infections within a population, each infection may be diagnosed with probability $\alpha \leqslant 1$, depending on the sensitivity of the test and the likelihood that an infected person consults a doctor. If each of the A_t infections is diagnosed independently of the others, then the number of diagnosed cases a_t follows a binomial distribution $a_t \sim \mathrm{Bin}(A_t, \alpha)$. Then $\langle a_t|A_t = j\rangle = \alpha j$ is given by the expected value of the binomial distribution. This implementation of subsampling conforms with the definition above, with the sampling probability α and the constant in (iii) being identical here.

2. Sampling a subset of system components. In a different application, assume a high-dimensional system of interacting units that forms the substrate on which activation propagates. Often, the states of a subset of units are observed continuously, for example by placing electrodes that record the activity of the same set of neurons over the entire recording (Fig. 1**b**). This implementation of subsampling in finite size systems is mathematically approximated as follows: If n out of all N model units are sampled, the probability to sample a_t active units out of the actual A_t active units follows a hypergeometric distribution, $a_t \sim \mathrm{Hyp}(N, n, A_t)$. As $\langle a_t|A_t = j\rangle = jn/N$, this representation satisfies Def. 1 with $\alpha = n/N$. Choosing this special implementation of subsampling allows to evaluate $\mathrm{Var}[a_t]$ further in terms of A_t:

$$\begin{aligned}\mathrm{Var}[a_t] &= \langle \mathrm{Var}[a_t|A_t]\rangle + \mathrm{Var}[\langle a_t|A_t\rangle] \\ &= n\langle \frac{A_t}{N}\frac{N-A_t}{N}\frac{N-n}{N-1}\rangle + \mathrm{Var}[\frac{n}{N}A_t] \\ &= \frac{1}{N}\frac{n}{N}\frac{N-n}{N-1}\left(N\langle A_t\rangle - \langle A_t^2\rangle\right) + \frac{n^2}{N^2}\mathrm{Var}[A_t] \\ &= \frac{n}{N^2}\frac{N-n}{N-1}\left(N\langle A_t\rangle - \langle A_t\rangle^2\right) + \left(\frac{n^2}{N^2} - \frac{n}{N^2}\frac{N-n}{N-1}\right)\mathrm{Var}[A_t]. \end{aligned} \tag{3}$$

This expression precisely determines the variance $\mathrm{Var}[a_t]$ under subsampling from the properties $\langle A_t\rangle$ and $\mathrm{Var}[A_t]$ of the full process (which for BPs are known from Lemma 1), and from the parameters of subsampling n and N. Using Eq. (3), we could predict the linear regression slopes $\hat{r}_k$ under subsampling (Theorem 5, Eq. (17)) in more detail:

$$r_k = \alpha^2\frac{\mathrm{Var}[A_t]}{\mathrm{Var}[a_t]}m^k = \frac{n(N-1)\mathrm{Var}[A_t]}{(N-n)(N\langle A_t\rangle - \langle A_t\rangle^2) + (nN-N)\mathrm{Var}[A_t]}m^k =: b(N, n, \langle A_t\rangle, \mathrm{Var}[A_t])\,m^k. \tag{4}$$

The term $b = b(N, n, \langle A_t\rangle, \mathrm{Var}[A_t])$ is constant when subsampling a given (stationary) system, and quantifies the factor by which $\hat{m}_C$ is biased when using the conventional estimate for m. It depends on N, n and the first two moments of A_t and is thus known for a BP. This relation was used for Fig. 1**c**.

Supplementary Note 4 MR estimation

We here derive an estimator for the mean offspring m based on the autoregressive representation of the BP,

$$\langle A_{t+1}|A_t = j\rangle = mj + h. \tag{5}$$

This novel estimator is based on multistep regressions[9] (MR estimator), which generalize (5) to arbitrary time steps k. From iteration of Eq. (5), it is easy to see that

$$\langle A_{t+k}|A_t = j\rangle = m^k j + h\frac{1-m^k}{1-m}. \tag{6}$$

Definition 2 (Multistep regression estimator). Consider a subsampled BP $\{a_t\}$ of length T. Let $k_{\max} \in \mathbb{N}$, $k_{\max} \geqslant 2$. Then multistep regression (of $k_{\max}$-th order) estimates m in the following way:

† Throughout this manuscript, the conditional random variable $(a_t|A_t = j)$ is to be read as "a_t given the realization $A_t = j$ of the random variable A_t".

1. For $k = 1, \dots, k_{\max}$, estimate the slope $\hat{r}_k$ and offset $\hat{s}_k$ of linear regression between the pairs $\{(a_t, a_{t+k})\}_{t=0}^{T-k}$, e.g. by least square estimation (Fig. 1**e**), i.e. by minimizing the residuals

$$R_k(\hat{r}_k, \hat{s}_k) = \sum_t (a_{t+k} - (\hat{r}_k \cdot a_t + \hat{s}_k))^2 . \tag{7}$$

2. Based on the relation[9] $r_k = b \cdot m^k$, estimate $\hat{b}$ and $\hat{m}$ by minimizing the sum of residuals

$$R(\hat{b}, \hat{m}) = \sum_{k=1}^{k_{\max}} \left(\hat{r}_k - \hat{b} \cdot \hat{m}^k\right)^2 , \tag{8}$$

with the collection of slopes $\{\hat{r}_k\}_{k=1}^{k_{\max}}$ obtained from step 1 (Fig. 1**f**).

Then $\hat{m}$ is the multistep regression (MR) estimate of the mean offspring m. For the application to experimental data, we further applied tests to identify nonstationarities (Supplementary Note 5).

We first prove that the MR estimator is consistent in the fully sampled case, and will then show the consistency under subsampling. First, we need the following result about the individual linear regression slopes $\hat{r}_k$ under full sampling:

Theorem 2. *The slope $\hat{r}_k$, obtained from A_t under full sampling, is a consistent estimator for m^k. If the process is subcritical, then the offset $\hat{s}_k$ is also a consistent estimator for $h\frac{1-m^k}{1-m}$.*

Remark. For $k = 1$, these results were already obtained by [8, 10, 11], and details can be found in these sources. Based on their proofs, we here show the generalization to k timesteps.

Proof. Let $k \in \mathbb{N}$, $i \in \{0, \dots, k-1\}$. Construct a new random process by starting at time i and taking every k-th time step of the original process A_t. This new process is given by $A_{t'}^{(k,i)} = A_{i+k \cdot t'}$ with the index $t' \in \mathbb{N}$. Hence, the "time" t' of this new process relates to the time t of the old process as $t = i + k \cdot t'$. For a time series of length T, let $\hat{r}^{(k,i)}$ be the least square estimator for the slope and $\hat{s}^{(k,i)}$ the least square estimator for the intercept of linear regression on all pairs $(A_{t'+1}^{(k,i)}, A_{t'}^{(k,i)})$ from the time series $\{A_{t'}^{(k,i)}\}_{t'=0}^{\lfloor (T-1)/k \rfloor}$. We will derive that $\hat{r}^{(k,i)}$ is a consistent estimator for m^k. According to [11], it is sufficient to show that the evolution of $A_{t'}^{(k,i)}$ can be rewritten as

$$A_{t'}^{(k,i)} = m^k \cdot A_{t'-1}^{(k,i)} + h\frac{1-m^k}{1-m} + \epsilon_{t'}^{(k,i)} \tag{9}$$

with a martingale difference sequence $\epsilon_{t'}^{(k,i)}$, as this is a stochastic regression equation. Hence, consider

$$\epsilon_{t'}^{(k,i)} = A_{t'}^{(k,i)} - m^k \cdot A_{t'-1}^{(k,i)} - h\frac{1-m^k}{1-m} = A_{i+kt'} - m^k \cdot A_{i+k\,(t'-1)} - h\frac{1-m^k}{1-m}. \tag{10}$$

We now show that $(\epsilon_{t'}^{(k,i)})_{t' \in \mathbb{N}}$ is a martingale difference sequence for all k. From iteration of Eq. (6), it is easy to see that

$$\langle A_{t'}^{(k,i)} | A_{t'-1}^{(k,i)} = j \rangle = \langle A_{kt'+i} | A_{kt'-k+i} = j \rangle = m^k j + h\frac{1-m^k}{1-m} \tag{11}$$

holds. Hence, $\langle \epsilon_{t'}^{(k,i)} \,|\, A_{t'-1}^{(k,i)} = j \rangle = 0$ for any j and $\{\epsilon_{t'}^{(k,i)}\}$ is indeed a martingale difference sequence. Therefore, $\{A_{t'}^{(k,i)}\}_{t'=0}^{\lfloor T/k \rfloor}$ satisfies a linear stochastic regression equation with slope m^k and intercept $h\frac{1-m^k}{1-m}$. The least square estimators return unbiased and consistent estimates for the slope and intercept in the subcritical case, i.e. the estimators converge in probability[8,10,11]:

$$\hat{r}^{(k,i)} \xrightarrow{\text{p}} m^k \qquad \hat{s}^{(k,i)} \xrightarrow{\text{p}} h\frac{1-m^k}{1-m}.$$

In the critical and supercritical cases, only $\hat{r}^{(k,i)} \xrightarrow{\text{p}} m^k$ holds following [11]. Hence, we obtain $\hat{r}_k \xrightarrow{\text{p}} m^k$ for all m and $\hat{s}_k \xrightarrow{\text{p}} h(1-m^k)/(1-m)$ if $m < 1$. □

Corollary 3. *As least square estimation of $\hat{b}$ and $\hat{m}$ from minimizing the residual* (8) *is consistent, multistep regression is a consistent estimator for m under full sampling, $\hat{m} \xrightarrow{\text{p}} m$.*

These results were obtained for BPs. However, the derivation is here only based on the autoregressive representation (5), motivation the following proposition:

Conjecture 4. *Multistep regression is a consistent estimator for m for any PAR satisfying Eq.* (5).

Numerical results for AR(1) and Kesten processes support this conjecture[9] (Supplementary Fig. 1).
Next, we show that MR estimation is consistent in the subcritical case even if only the subsampled a_t is known:

Theorem 5. *Let A_t be a PAR with $m < 1$ and a stationary limiting distribution A_∞ and let the PAR be started in the stationary distribution, i.e. $A_0 \sim A_\infty$. Let a_t be a subsampling of A_t. Multistep regression (MR) on the subsampled a_t is a consistent estimator of the mean offspring m.*

Proof. The existence of a stationary distribution A_∞ was shown by [2]. The least square estimator for the slope of linear regression is also given by[12]

$$\hat{r}_k = \hat{\rho}_{a_t a_{t+k}} \frac{\hat{\sigma}_{a_t}}{\hat{\sigma}_{a_{t+k}}} \tag{12}$$

with the the estimated standard deviations $\hat{\sigma}_{a_t}$ and $\hat{\sigma}_{a_{t+k}}$ of a_t and a_{t+k} respectively. In the subcritical state, $\sigma_{a_t} = \sigma_{a_{t+k}}$ because of stationarity. Thus estimating the linear regression slope is equivalent to estimating the Pearson correlation coefficient $\hat{\rho}_{a_t a_{t+k}} = \hat{\rho}_{a_t}(k)$ (which is identical to the autocorrelation function of a_t). In the following, we calculate the Pearson correlation coefficient for the subsampled time series by evaluating $\langle a_t a_{t+k} \rangle$. We use the law of total expectation in order to express $\langle a_t a_{t+k} \rangle$ not in dependence of a_t, but in terms of A_t:

$$\langle a_t a_{t+k} \rangle = \langle \langle a_t a_{t+k} \,|\, A_t, A_{t+k} \rangle \rangle_{A_{t+k}, A_t}, \tag{13}$$

where the inner expectation value is taken with respect to the joint distribution of a_{t+k} and a_t, and the outer with respect to the joint distribution of A_{t+k} and A_t. Through conditioning on both A_t and A_{t+k}, $(a_t \,|\, A_t)$ and $(a_{t+k} \,|\, A_{t+k})$ become independent due to Def. 1. Hence, the joint distribution of $(a_t, a_{t+k} \,|\, A_t, A_{t+k})$ factorizes, and the expectation value factorizes as well. By definition, $\langle a_t \,|\, A_t = j \rangle = \alpha j + \beta$ and hence

$$\langle a_t a_{t+k} \rangle = \langle (\alpha A_{t+k} + \beta)(\alpha A_t + \beta) \rangle_{A_{t+k}, A_t} \tag{14}$$

Without loss of generality, we here show the proof for $\beta = 0$ which is easily extended to the general case. We express $\langle a_t a_{t+k} \rangle$ in terms of Eq. (6) using the law of total expectation again:

$$\begin{aligned}
\langle a_t a_{t+k} \rangle &= \alpha^2 \langle A_t A_{t+k} \rangle \\
&= \alpha^2 \langle \langle A_t A_{t+k} \,|\, A_t \rangle \rangle_{A_t} \\
&= \alpha^2 \langle A_t \left(m^k A_t + h \frac{1 - m^k}{1 - m} \right) \rangle_{A_t} \\
&= \alpha^2 \left(m^k \langle A_t^2 \rangle + (1 - m^k) \langle A_t \rangle^2 \right),
\end{aligned}$$

where the first expectation was taken with respect to the joint distribution of A_t and A_{t+k}. We then used that $\langle A_t^2 \rangle$ and $\langle A_t \rangle = h/(1 - m)$ exist, which follows from stationarity of the process. By a similar argument,

$$\langle a_{t+1} \rangle = \langle a_t \rangle = \langle \langle a_t \,|\, A_t \rangle \rangle_{A_t} = \alpha \langle A_t \rangle = \alpha \frac{h}{1 - m} \tag{15}$$

and combining these results the covariance is

$$\mathrm{Cov}[a_{t+k}, a_t] = \langle a_{t+k} a_t \rangle - \langle a_{t+k} \rangle \langle a_t \rangle = \alpha^2 \left(m^k \langle A_t^2 \rangle + (1 - m^k) \langle A_t \rangle^2 \right) - \alpha^2 \langle A_t \rangle^2 = \alpha^2 m^k \mathrm{Var}[A_t]. \tag{16}$$

Therefore, we find that the estimator $\hat{r}_k$ converges in probability:

$$\hat{r}_k \xrightarrow{\mathrm{p}} \rho_{a_t a_{t+k}} = \frac{\mathrm{Cov}[a_{t+k}, a_t]}{\mathrm{Var}[a_t]} = \alpha^2 \frac{\mathrm{Var}[A_t]}{\mathrm{Var}[a_t]} m^k. \tag{17}$$

Hence, the bias of of the conventional estimator $\hat{m}_C = \hat{r}_1$ is precisely given by the factor $b = \alpha^2 \mathrm{Var}[A_t]/\mathrm{Var}[a_t]$. However, importantly the relation $\hat{r}_k = \hat{b}\,\hat{m}^k$ still holds for the subsampled a_t. Given a collection of multiple linear regressions $\hat{r}_1, \ldots, \hat{r}_{k_{\max}}$, the least square estimation of $\hat{b}$ and $\hat{m}$ from minimizing the residual (8) yields a consistent estimator $\hat{m}$ for the mean offspring m even under subsampling and only requires the knowledge of a_t. □

This proof also showed that the conventional estimator[8] is biased under subsampling:

Corollary 6. *Let $\{a_t\}$ be a subsampling of a subcritical PAR $\{A_t\}$. Then the conventional linear regression estimator $\hat{m}_C = \hat{r}_1$ by [8] is biased by $m(\alpha^2 \frac{\mathrm{Var}[A_t]}{\mathrm{Var}[a_t]} - 1)$. Equivalently, it is biased by the factor $\alpha^2 \frac{\mathrm{Var}[A_t]}{\mathrm{Var}[a_t]}$.*

Nonstationarity, criticality and supercriticality. The consistency of the estimator in the fully sampled case is included in our proof of Lemma 2 and follows from the results by [8, 11]. Our proof for the subsampled case (Theorem 5), in contrast, strictly requires stationarity ($A_t \sim A_\infty$ for any t) and the existence of the first two moments of A_t. We expect that the MR estimator is also consistent if the subcritical process is not started in the stationary distribution, $A_0 \nsim A_\infty$, because the results by [2] show that it will converge to this stationary distribution as $t \to \infty$ (Supplementary Fig. 2). Furthermore, numerical results suggest that the MR estimator is also consistent for critical and supercritical cases, where no stationary distribution exists (Fig. 3**d**).

Supplementary Note 5 Identifying common non-stationarities and Poisson activity.

In many types of analyses, non-stationarities in the time series can lead to wrong results, typically an overestimation of $\hat{m}$. We developed tests to exclude data sets with signatures of common non-stationarities. The different non-stationarities, their impact on the r_k and the rules for rejection of time series are outlined below.

First, *transient* increases of the drive h_t, e.g. in response to a stimulus, lead to a transient increase in $\langle A_t \rangle$. These transients induce correlations or anti-correlations, which prevail on long time scales (Supplementary Fig. 3**c**,**d**). The autocorrelation function is therefore better captured by an exponential with offset, $r_k = b_{\text{offset}} \cdot m_{\text{offset}}^k + c_{\text{offset}}$. If the residual of this exponential with offset R^2_{offset} was smaller than the residual of the MR model R^2_{exp} by a factor of two, $H_{\text{offset}} = (2 \cdot R^2_{\text{offset}} < R^2_{\text{exp}})$, then the data set was rejected. The factor two punishes for the differences in degree of freedom: The residuals of a model with two free parameters (exponential with offset) instead of one (exponential only) can only be smaller.

Second, ramping of the drive can lead to overestimation of m (Supplementary Fig. 3**e**). The comparison of the two models with and without offset introduced above serves as a consistency check able to identify ramping: if the data are captured by a BP, both models should infer identical $\hat{m}$. Thus, a difference between $\hat{m}_{\text{exp}}$ and $\hat{m}_{\text{offset}}$ hints at the invalidity of MR estimation. Instead of $\hat{m}$, we compared the autocorrelation times $\hat{\tau}_{\text{offset}} = -\Delta t / \log \hat{m}_{\text{offset}}$ and $\hat{\tau}_{\text{exp}}$ obtained from both models, as the logarithmic scaling increases the sensitivity. If their relative difference was too large, then the data are inconsistent with a BP and MR estimation is invalid: $H_\tau = (|\tau_{\text{exp}} - \tau_{\text{offset}}| / \min\{\tau_{\text{exp}}, \tau_{\text{offset}}\} > 2)$.

Third, when a system changes between different states of activity, e.g. up and down states, the drive rate $\langle h_t \rangle$ may experience sudden jumps. These can lead to spurious autocorrelation (Supplementary Fig. 3**f**). To identify these trends resulting from non-stationary input h_t or from choosing too short data sets, we tested whether the sequence of r_k was fit better by a linear regression $r_k = q_1 k + q_2$ on the pairs (k, r_k), than by the exponential relation (8). If the residuals R^2_{lin} were smaller than R^2_{exp}: $H_{\text{lin}} = (R^2_{\text{lin}} < R^2_{\text{exp}})$, data were rejected.

Apart from non-stationarities, even Poisson activity ($m = 0$, $A_t = h_t$) with stationary rate may lead to a spurious overestimation of $\hat{m}$ as well: for *subsampled* branching processes of *finite* duration, the Poisson case and processes close to criticality ($m = 1$) can show very similar autocorrelation results, because the sequence of r_k is expected to be absolutely or almost flat, respectively. Moreover, for $m = 0$ any solution on the manifold with $b = 0$ minimizes the residuals in Eq. (8). Hence, the estimator for $\hat{m}$ may yield any value depending on the initial conditions of the minimization scheme. To distinguish between $m = 0$ and $m > 0$, we used the fact that for $m = 0$, all slopes r_k are expected to be distributed around zero, $\langle r_k \rangle = 0$. In contrast, for processes with $m > 0$, all slopes are expected to be larger than zero $\langle r_k \rangle = b \cdot m^k > 0$. Thus to identify stationary Poisson activity, we tested (using a one-sided t-test) if the slopes obtained from the data were significantly larger than zero, yielding the p-value $p_{\bar{r} \leqslant 0}$ and the following test (Supplementary Fig. 3**b**): $H_{\bar{r} \leqslant 0} = (p_{\bar{r} \leqslant 0} \geqslant 0.1)$. The choice of the significance level should be guided by the severity of type I or II errors here: if it is set too liberal, Poisson activity may be mistaken for correlated activity, potentially even close-to-critical. On the other hand, if the significance level is too conservative, activity with long autocorrelation times may be spuriously considered Poissonian under strong subsampling (when b is small and all slopes only slightly differ from zero). For this study, we chose a significance level of $p_{\bar{r} \leqslant 0} < 0.1$ in order to not underestimate the risk of large activity cascades. To confirm candidates for Poisson activity identified through positive $H_{\bar{r} \leqslant 0}$, we assured that the r_k did not show a systematic trend, i.e. that linear regression of r_k as a function of k (see H_{lin} above) yielded slope zero: $H_{q_1=0} = (p_{q_1=0} \geqslant 0.05)$. The according significance level for this two sided test is then given by $p_{q_1 \neq 0} < 0.05$.

We discriminate the following cases in the order indicated in Supplementary Table 1: $\hat{m}$ obtained from MR estimation is only valid if none of the tests (except $H_{q_1=0}$, which is ignored here) is positive. A positive result for any of H_{offset}, H_τ, or H_{lin} indicates non-stationarities, the data are not explained by a stationary BP, and MR estimation is invalid. If $H_{\bar{r} \leqslant 0}$ is positive, the data are potentially consistent with Poisson activity ($m = 0$). This is only the case if $H_{q_1=0}$ is also positive. If otherwise $H_{q_1=0}$ is negative, the Poisson hypothesis is also rejected and MR estimation invalid. This strategy correctly identified the validity of MR estimation for all investigated cases: stationary BPs with $m = 0.98$ and $m = 0.0$ were accepted, while nonstationary BPs with transient changes, ramping, or sudden jumps of the drive were excluded (Supplementary Fig. 3).

Supplementary Note 6 Variance of the estimates.

The distribution of $\hat{m}$ is consistent with a normal distribution $\mathcal{N}(m, \sigma^2_{\hat{m}})$ centered around the true mean offspring m (Supplementary Fig. 4**a**; numerical results). The variance $\sigma^2_{\hat{m}}$ depends on the branching ratio m, the mean activity $\langle A_t \rangle$, the length L of the time series, and the sampling fraction α. Each of these factors affects $\sigma^2_{\hat{m}}$ mainly by changing the *effective length* of the time series, i.e. the number of non-zero entries $l = |\{A_t | A_t > 0\}|$. Thus, regardless of the actual time series length L or the mean activity $\langle A_t \rangle$, the variance scales as a power-law in l, $\text{Var}[\hat{m}] \propto l^{-\gamma}$ (Supplementary Fig. 4**b**). The exponent of this power-law depends on m.

The closer to criticality the process is, the larger the exponent γ, i.e. the larger the benefit from longer time series length l. For $m = 0.99$, we found $\gamma \approx 3/2$. The performance of the estimator is in principle independent of the mean activity: Small $\langle A_t \rangle$ only affect the variance of the MR estimator through a potential decrease of l.

Similarly, the degree of subsampling only affects the variance of the estimator through a decrease of the effective length of a_t. While there may be a significant rise in $\sigma^2_{\hat{m}}$ when reducing the sampling fraction α, this increase can be explained by the coincidental decrease in l, as the rescaled variance $\sigma^2_{\hat{m}} \cdot l^{\gamma}$ remains within one order of magnitude over four decades of the sampling fraction α (Supplementary Fig. 4**c**).

How does the variance change close to the critical transition? We found that the answer to this question highly depends on the specific choice of the parameters: if m is varied, one can either keep $\langle A_t \rangle$ or h constant, not both at the same time. If the mean activity $\langle A_t \rangle$ is fixed by choosing $h = \langle A_t \rangle (1 - m)$, then the variance of the process scales as $\mathrm{Var}[A_t] \propto 1/(1-m)$ (Theorem 1). As $m \to 1$, the activity will inevitably get into a regime, where bursts of activity ($A_t > 0$) are disrupted by intermittent quiescent periods (A_t), thereby reducing l. In turn, the variance of the estimator increases as detailed before.

If however, the drive h is kept constant, we found that the variance scales linearly in the distance to criticality $\epsilon = 1 - m$ over at least 5 orders of magnitude of ϵ: $\sigma^2_{\hat{m}} \propto \epsilon$ (Supplementary Fig. 4**d**). Thus, the variance decreases when approaching criticality, while the relative variance $\sigma^2_{\hat{m}}/\epsilon$ is constant. Note, however, that even though the standard deviation also decreases when approaching criticality ($\sigma_{\hat{m}} \propto \sqrt{\epsilon}$), the relative standard deviation increases ($\sigma_{\hat{m}}/\epsilon \propto 1/\sqrt{\epsilon}$).

For other measures of variation (e.g. quadratic (like the mean squared error MSE) and linear (like the inter-quartile range IQR)), we obtained scaling laws with the same exponents.

Confidence interval estimation. We used a model based approach to estimate confidence intervals for both simulation and experimental data (for Figs. 1**c**,**d**, 2**c**,**d**, and 3**d**), because classical bootstrapping methods underestimate the estimator variance by treating all slopes r_k independently, while they are in fact dependent. We found that our model based approach constructs more conservative and representative confidence intervals.

For simulations, we simulated $B \in \mathbb{N}$ independent copies of the investigated model and applied MR estimation to each copy, yielding a collection of B independent estimates $\{\hat{m}^{(b)}\}_{b=1}^{B}$.

For experimental time series a_t with length L, mean activity $\langle a_t \rangle$, and number of sampled units n, MR estimation yields an estimate $\hat{m}$. We then simulated B copies of branching networks $\{A_t^{(b)}\}_{b=1}^{B}$ (for simulation details see Supplementary Note 8) with $N = 10{,}000$ units, $m = \hat{m}$ as inferred by MR estimation, and length L and rate $\langle a_t \rangle$ to match the data. The rate was matched by setting the drive to $h = \langle a_t \rangle (1 - \hat{m}) N/n$. Thereby, after subsampling n units, the mean activity of each resulting time series $a_t^{(b)}$ matched that of the original time series a_t, $\langle a_t^{(b)} \rangle = \langle a_t \rangle$. This procedure gives B copies of a BN that all match a_t in terms of the mean activity, the branching ratio, time series length, and number of sampled units. Applying MR estimation to these BNs yields a collection of B independent estimates $\{\hat{m}^{(b)}\}_{b=1}^{B}$. For both simulation and experimental data, the distribution of $\hat{m}$ and confidence intervals can be constructed from this collection.

Supplementary Note 7 Expectation maximization based on Kalman filtering

Kalman filtering is a method to predict the original time series A_t given a measurement a_t, defined for AR(1) processes and affine measurement transformation

$$\begin{aligned} A_{t+1} &= m \cdot A_t + h_t \\ a_t &= \alpha \cdot A_t + \beta_t \end{aligned} \tag{18}$$

where h_t and β_t are independent Gaussian random variables $h_t \sim \mathcal{N}(h, \xi^2)$ and $\beta_t \sim \mathcal{N}(\beta, \zeta^2)$ and m and α constant real numbers. Assuming that $A_0 \sim \mathcal{N}(A, \psi)$, Kalman filtering infers the original time series $A_t | a_t, \mathscr{M}$ given a measured time series a_t and the known model $\mathscr{M} = (m, h, \xi^2, \alpha, \beta, \zeta^2, A, \psi)$. Based on an iterative expectation maximization algorithm which incorporates Kalman filtering[13–15], the model parameters $\mathscr{M}$ can be estimated from a time series a_t. We used this algorithm to infer m. Because of the mutual dependence of the model parameters, we also needed to infer h, ξ^2, α, β, and ζ^2. In order to reduce the dimensionality of the maximization step, we disregarded A and ψ, as the influence of the initial value decreases if the time series gets long. For initial values, we chose $m = 0.5$ in the center of the range of interest for m, $h_t = \langle a_t \rangle \cdot (1 - m)$ (see Supplementary Note 2), $\xi = 0.1 \cdot h_t$, $\alpha = 1$, $\beta = 0$, and $\zeta = 0.1$. We further chose $A = \langle a_t \rangle$ and $\psi^2 = \mathrm{Var}[a_t]$ for the two model parameters that were not optimized.

We considered two termination criteria for the EM algorithm: First, it is recommended to restrict the EM algorithm to 10 – 20 cycles in order to avoid overfitting, a common problem with likelihood-based fitting methods for multidimensional model parameters. Therefor we considered $\hat{m}$ inferred after 20 EM cycles. Second, we considered $\hat{m}$ after the results of two subsequent EM cycles did not differ by more than 0.01%.

We used the publicly available Python implementation of the Kalman EM algorithm, *pykalman*. All parameters were chosen as detailed above. The analysis was performed on a computer cluster, and reached runtimes of several days up to projected runtimes of weeks. In fact, this computational demand was a limiting factor in terms of widespread application. In contrast, MR estimation terminated within half a second on the same CPUs.

Supplementary Note 8 Simulations

Branching process. We simulated BPs according to Eq. (1) in the following way: Realizations of the random numbers $y_{t,i}$ and h_t describing the number of offsprings, and the drive, were each drawn from a Poisson distribution: $y_{t,i} \sim \mathrm{Poi}(m)$ with mean m, and $h_t \sim \mathrm{Poi}(h)$ with mean h, respectively. Here, we used Poisson distributions as they allow for non-trivial offspring distributions with easy control of the branching ratio m by only one parameter. For the brain, one might assume that each neuron is connected to k postsynaptic neurons, each of which is excited with probability p, motivating a binomial offspring distribution with mean $m = kp$. As in cortex k is typically large and p is typically small, the Poisson limit is a reasonable approximation. For the performance of the MR estimator and the limit behavior of the BP, the particular form of the law Y is not important such that the special choice we made here does not restrict the generality of our results.

The mean rate $\langle A_t \rangle$ depends on m and h (Lemma 1). In the simulation we varied m and fixed $\langle A_t \rangle = 100$ by adjusting h accordingly if not stated otherwise. For subsampling the BP, each unit is observed independently with probability $p \leqslant 1$. Then a_t is distributed following a binomial distribution $\mathrm{Bin}(A_t, p)$, and subsampling is implemented by drawing a_t from A_t at each time step. As $\langle a_t \rangle = p\, A_t$, this implementation of subsampling satisfies the definition of stochastic subsampling with $\alpha = p$, $\beta = 0$.

Branching network. In addition to the classical branching process, we also simulated a branching network model (BN) by mapping a branching process[1,16] onto a fully connected network of $N = 10,000$ neurons. An active neuron activated each of its k postsynaptic neurons with probability $p = m/k$. Here, the activated postsynaptic neurons were drawn randomly without replacement at each step, thereby avoiding that two different active neurons would both activate the same target neuron. Similar to the BP, the BN is critical for $m = 1$ in the infinite size limit, and subcritical (supercritical) for $m < 1$ ($m > 1$). As detailed for the BP, h was adjusted to the choice of m to achieve $\langle A_t \rangle = 100$, which corresponds to a rate of 0.01 spikes per neuron and time step. Subsampling[17] was applied to the model by sampling the activity of n neurons only, which were selected randomly before the simulation, and neglecting the activity of all other neurons.

Self-organized critical model. The SOC neural network model we used here is the Bak-Tang-Wiesenfeld (BTW) model[18]. Translated to a neuroscience context, the model consisted of $N = 10,000$ (100×100) non-leaky integrate and fire neurons. A neuron i spiked if its membrane voltage $V_i(t)$ reached a threshold θ:

$$\text{If } V_i(t) > \theta,\ V_i(t+1) = V_i(t) - 4. \tag{19}$$

Note that the choice of θ does not change the activity of the model at all, so we set $\theta = 0$ for convenience. The model neurons were arranged on a 2D lattice, and each neuron was connected locally to its four nearest neighbors with coupling strength $\alpha_{ij} = \alpha$:

$$V_i(t+1) = V_i(t) + \sum_j \alpha_{ij}\delta(t - T_j) + h_i(t), \tag{20}$$

where T_j denotes the spike times of neuron j, and $h_i(t)$ is the Poisson drive to neuron i with mean rate h as defined for the BP above. Note that the neurons at the edges and corners of the grid had only 3 and 2 neighbors, respectively. This model is equivalent to the well-known Bak-Tang-Wiesenfeld model[18] if $h \to 0$ and $\alpha = 1$. Subsampling[17] was implemented in the same manner as for the BN.

Parameter choices. If not stated otherwise, simulations were run for $L = 10^7$ time steps or until A_t exceeded 10^9, i.e. approximately half of the 32 bit integer range. If not stated otherwise, confidence intervals (Supplementary Note 6) were estimated from $B = 100$ samples, both for simulation and experiments.

In Figs. 1**c**,**d**, BNs and the BTW model were simulated with $N = 10^4$ units and $\langle A_t \rangle = 100$. In Fig. 1**e**, BPs were simulated with $m = 0.9$ and $\langle A_t \rangle = 100$.

In Fig. 3**c**, subcritical and critical BNs with $N = 10^4$ and $\langle A_t \rangle = 100$ were simulated, and $n = 100$ units sampled. Because of the non-stationary, exponential growth in the supercritical case, here BPs were simulated with $h = 0.1$ and units observed with probability $\alpha = 0.01$.

Supplementary Note 9 Epidemiological recordings

WHO data on measles worldwide. Time series with yearly case reports for measles in 194 different countries are available online from the World Health Organization (WHO) for the years between 1980 and 2014. MR estimation was applied to these time series. Because they contain very few data points and potential long-term drifts, we applied the consistency checks detailed above for every country (Supplementary Table 1). After these checks, 124 out of the 194 surveyed countries were accepted for MR analysis and included in our analysis. Yearly information on approximate vaccination percentages (measles containing vaccine dose 1, MCV1) for the same countries and time span are also available online from the WHO.

RKI data on norovirus, measles and MRSA in Germany. For Germany, the Robert-Koch-Institute (RKI) surveys a range of infectious diseases on a weekly basis, including measles, norovirus, and invasive meticillin-resistant Staphylococcus aureus (MRSA). Case reports are available through their SURVSTAT@RKI server[19]. Because of possible changes in report policies in the beginning of surveillance, we omitted the data from the first 6 months of each recording. Moreover, we omitted the incomplete week on the turn of the year, thus evaluating 52 full weeks in each year.

The MRSA recording showed a slow, small variation in the case reports that can be attributed to slow changes in the drive rates. To compensate for these slow drifts, we corrected the time series by subtracting a moving average over 3 years (156 weeks). We then applied MR estimation to the obtained time series. The recordings for measles and norovirus showed strong seasonal fluctuations of the case reports, resulting in a baseline oscillation of the autocorrelation function. We therefore used a modified model

$$r_k = b \cdot m^k + c \cdot \cos(2\pi k/T) \tag{21}$$

with a fixed period of $T = 52$ weeks, and estimated $\hat{m}$, $\hat{b}$, and $\hat{c}$ from minimizing the residual of this modified equation.

In order to obtain the naive estimates using the conventional linear regression estimator $\hat{m}_C = \hat{r}_1$, we used the following correction for seasonal fluctuations. Each incidence count a_t was normalized by the incidence counts from the same week, averaged over all years of recording ($\bar{a}_w = \langle a_{w+52\cdot y}\rangle_y$ with the average taken over the years y for any week $w = 1, \ldots, 52$), yielding the deseasonalized time series $a'_t = a_t/\bar{a}_{t \bmod 52}$. Linear regression was performed on this time series a'_t.

For Fig. 2**d**, subsampling was applied to the original time series assuming that every infection is diagnosed and reported with a probability α, yielding the binomial subsampling described in Supplementary Note 3. MR estimates were obtained from this subsampled time series according to Eq. (21), for the conventional estimator the subsampled time series was processed as described above.

Supplementary Note 10 Animal experiments

We evaluated spike population dynamics from recordings in rats, cats and monkeys. The rat experimental protocols were approved by the Institutional Animal Care and Use Committee of Rutgers University[20,21]. The cat experiments were performed in accordance with guidelines established by the Canadian Council for Animal Care[22]. The monkey experiments were performed according to the German Law for the Protection of Experimental Animals, and were approved by the Regierungspräsidium Darmstadt. The procedures also conformed to the regulations issued by the NIH and the Society for Neuroscience. The spike recordings from the rats and the cats were obtained from the NSF-founded CRCNS data sharing website[20–23].

In rats the spikes were recorded in CA1 of the right dorsal hippocampus during an open field task. We used the first two data sets of each recording group (ec013.527, ec013.528, ec014.277, ec014.333, ec015.041, ec015.047, ec016.397, ec016.430). The data-sets provided sorted spikes from 4 shanks (ec013) or 8 shanks (ec014, ec015, ec016), with 31 (ec013), 64 (ec014, ec015) or 55 (ec016) channels. We used both, spikes of single and multi units, because knowledge about the identity and the precise number of neurons is not required for the MR estimator. More details on the experimental procedure and the data-sets proper can be found in [20, 21].

For the spikes from the cat, neural data were recorded by Tim Blanche in the laboratory of Nicholas Swindale, University of British Columbia[22]. We used the data set pvc3, i.e. recordings in area 18 which contain 50 sorted single units[23]. We used that part of the experiment in which no stimuli were presented, i.e., the spikes reflected spontaneous activity in the visual cortex of the anesthetized cat. Because of potential non-stationarities at the beginning and end of the recording, we omitted data before 25 s and after 320 s of recording. Details on the experimental procedures and the data proper can be found in [22, 23].

The monkey data are the same as in [24, 25]. In these experiments, spikes were recorded simultaneously from up to 16 single-ended micro-electrodes ($\varnothing = 80\,\mu$m) or tetrodes ($\varnothing = 96\,\mu$m) in lateral prefrontal cortex of three trained macaque monkeys (M1: 6 kg ♀; M2: 12 kg ♂; M3: 8 kg ♀). The electrodes had impedances between 0.2 and 1.2 MΩ at 1 kHz, and were arranged in a square grid with inter electrode distances of either 0.5 or 1.0 mm. The monkeys performed a visual short term memory task. The task and the experimental procedure is detailed in [24]. We analyzed spike data from 12 experimental sessions comprising almost 12.000 trials (M1: 4 sessions; M2: 5 sessions; M3: 3 sessions). 6 out of 12 sessions were recorded with tetrodes. Spike sorting on the tetrode data was performed using a Bayesian optimal template matching approach as described in [26] using the "Spyke Viewer" software[27]. On the single electrode data, spikes were sorted with a multi-dimensional PCA method (Smart Spike Sorter by Nan-Hui Chen).

Analysis. For each recording, we collapsed the spike times of all recorded neurons into one single train of population spike counts a_t, where a_t denotes how many neurons spiked in the tth time bin Δt. We used $\Delta t = 4$ ms, reflecting the propagation time of spikes from one neuron to the next. Note that m scales with the bin size (bs) as $m(\text{bs} = k\Delta t) = m(\text{bs} = \Delta t)^k$, while the corresponding autocorrelation times are invariant under bin size changes. For Fig. 3**b** and Supplementary Fig. 6, we investigated single neuron activity by applying similar binning to the spike times of each neuron individually.

From these time series, we estimated $\hat{m}$ using the MR estimator with $k_{\max} = 2500$ (corresponding to 10 s) for the rat recordings, $k_{\max} = 150$ (600 ms) for the cat recording, and $k_{\max} = 500$ (2000 ms) for the monkey recordings, assuring that $k_{\max}$ was always in

the order of multiple autocorrelation times. Experiments were excluded if the tests according to Supplementary Note 5 detected potential nonstationarities.

Supplementary References

1. Harris, T. E. *The Theory of Branching Processes* (Springer Berlin, 1963).
2. Heathcote, C. R. A Branching Process Allowing Immigration. *J. R. Stat. Soc. Ser. B* **27**, 138–143 (1965).
3. Pakes, A. G. Branching Processes with Immigration. *J. Appl. Probab.* **8**, 32 (1971).
4. Ispány, M. & Pap, G. in *Lect. Notes Stat.* (eds Velasco, M. G., Puerto, I. M., Martínez, R., Molina, M., Mota, M. & Ramos, A.) 135–146 (Springer Berlin Heidelberg, 2010).
5. Alzaid, A. A. & Al-Osh, M. An Integer-Valued pth-Order Autoregressive Structure (INAR(p)) Process. *J. Appl. Probab.* **27**, 314 (1990).
6. Kachour, M. & Yao, J. F. First-order rounded integer-valued autoregressive (RINAR(1)) process. *J. Time Ser. Anal.* **30**, 417–448 (2009).
7. Kesten, H. Random difference equations and Renewal theory for products of random matrices. *Acta Math.* **131**, 207–248 (1973).
8. Heyde, C. C. & Seneta, E. Estimation Theory for Growth and Immigration Rates in a Multiplicative Process. *J. Appl. Probab.* **9**, 235 (1972).
9. Statman, A., Kaufman, M., Minerbi, A., Ziv, N. E. & Brenner, N. Synaptic Size Dynamics as an Effectively Stochastic Process. *PLoS Comput. Biol.* **10**, e1003846 (2014).
10. Venkataraman, K. N. A Time Series Approach to the Study of the Simple Subcritical Galton-Watson Process with Immigration. *Adv. Appl. Probab.* **14**, 1–20 (1982).
11. Wei, C. & Winnicki, J. Estimation of the Means in the Branching Process with Immigration. *Ann. Stat.* **18**, 1757–1773 (1990).
12. Kenney, J. F. & Keeping, E. S. Linear regression and correlation. *Math. Stat.* **1**, 252–285 (1962).
13. Hamilton, J. D. *Time series analysis* (Princeton university press Princeton, 1994).
14. Shumway, R. H. & Stoffer, D. S. An approach to time series smoothing and forecasting using the EM algorithm. *J. Time Ser. Anal.* **3**, 253–264 (1982).
15. Ghahramani, Z. & Hinton, G. E. *Parameter estimation for linear dynamical systems* (Technical Report, University of Toronto, 1996).
16. Haldeman, C. & Beggs, J. Critical Branching Captures Activity in Living Neural Networks and Maximizes the Number of Metastable States. *Phys. Rev. Lett.* **94**, 058101 (2005).
17. Priesemann, V., Munk, M. H. J. & Wibral, M. Subsampling effects in neuronal avalanche distributions recorded in vivo. *BMC Neurosci.* **10**, 40 (2009).
18. Bak, P., Tang, C. & Wiesenfeld, K. Self-organized criticality: An explanation of the 1/f noise. *Phys. Rev. Lett.* **59**, 381–384 (1987).
19. Robert-Koch-Institute. *SurvStat@RKI 2.0*
20. Mizuseki, K., Sirota, A., Pastalkova, E. & Buzsáki, G. *Multi-unit recordings from the rat hippocampus made during open field foraging* 2009.
21. Mizuseki, K., Sirota, A., Pastalkova, E. & Buzsáki, G. Theta Oscillations Provide Temporal Windows for Local Circuit Computation in the Entorhinal-Hippocampal Loop. *Neuron* **64**, 267–280 (2009).
22. Blanche, T. *Multi-neuron recordings in primary visual cortex* 2009.
23. Blanche, T. J. & Swindale, N. V. Nyquist interpolation improves neuron yield in multiunit recordings. *J. Neurosci. Methods* **155**, 81–91 (2006).
24. Pipa, G., Städtler, E. S., Rodriguez, E. F., Waltz, J. A., Muckli, L. F., Singer, W., Goebel, R. & Munk, M. H. J. Performance- and stimulus-dependent oscillations in monkey prefrontal cortex during short-term memory. *Front. Integr. Neurosci.* **3**, 25 (2009).
25. Priesemann, V., Wibral, M., Valderrama, M., Pröpper, R., Le Van Quyen, M., Geisel, T., Triesch, J., Nikolić, D. & Munk, M. H. J. Spike avalanches in vivo suggest a driven, slightly subcritical brain state. *Front. Syst. Neurosci.* **8**, 108 (2014).
26. Franke, F., Natora, M., Boucsein, C., Munk, M. H. J. & Obermayer, K. An online spike detection and spike classification algorithm capable of instantaneous resolution of overlapping spikes. *J. Comput. Neurosci.* **29**, 127–48 (2010).
27. Pröpper, R. & Obermayer, K. Spyke Viewer: a flexible and extensible platform for electrophysiological data analysis. *Front. Neuroinform.* **7**, 26 (2013).
